# Habitat and Trophic Specialization Among Greenland Cod (*Gadus ogac*) Morphotypes in the Context of Climate Change Resilience

**DOI:** 10.1002/ece3.71908

**Published:** 2025-09-10

**Authors:** Stephanie Chan, Harri Pettitt‐Wade, Jack P. W. Hollins, Tristan Pearce, Lisa Loseto, Teah G. Burke, Nigel E. Hussey

**Affiliations:** ^1^ Natural Resources and Environmental Studies University of Northern British Columbia Prince George British Columbia Canada; ^2^ Department of Ecosystem Science and Management University of Northern British Columbia Prince George British Columbia Canada; ^3^ Department of Integrative Biology University of Windsor Windsor Ontario Canada; ^4^ Freshwater Institute, Fisheries and Oceans Canada Winnipeg Manitoba Canada; ^5^ Department of Geography, Earth, and Environmental Sciences University of Northern British Columbia Prince George British Columbia Canada; ^6^ Centre for Earth and Observation Science (CEOS), Department of Environment and Geography University of Manitoba Winnipeg Manitoba Canada

**Keywords:** Canadian Arctic, Greenland cod, individual specialization, morphometrics, stable isotopes

## Abstract

Morphological variation is observed in many fish species; however, the direct ecological consequences of this variation in terms of specialists or generalists in resource use are rarely studied. Understanding the degree of specialist or generalist behavior among morphotypes has the potential to provide insight into the ability of Arctic fish species to adapt to ongoing climate change. Here, we estimated morphological variation and habitat‐trophic metrics from carbon (*δ* ^13^C) and nitrogen (*δ* ^15^N) stable isotopes in Greenland cod ( *Gadus ogac* ) collected along the marine coast near Ulukhaktok, Northwest Territories (NT), in the western Canadian Arctic (*n* = 45). Principal component analysis (PCA) of linear morphometric measurements and subsequent k‐means clustering categorized fish into two morphological groups driven primarily by head shape and body depth. Mean *δ* ^13^C and *δ* ^15^N values did not differ significantly between morphological groups; however, measures of individual specialization showed that the morphotype with the smaller head and slender body had lower habitat specialization and higher trophic specialization compared to the morphotype with the larger head and stockier body. This observed gradient suggests that morphotype‐specific behaviors can be observed over a generalist‐specialist gradient rather than as distinct groups and may benefit generalist populations in the future due to their ability to undergo resource shifts. The integrated approach used here informs our understanding of species' flexibility to competition and food web shifts with ongoing borealization. The findings highlight the importance of considering individual‐level data and the degree to which a population exhibits specialization‐generalization in fisheries co‐management in the Arctic.

## Graphical abstract

Morphological variation and habitat‐trophic metrics from carbon (*δ* ^13^C) and nitrogen (*δ* ^15^N) stable isotopes were estimated in Greenland cod ( *Gadus ogac* ) collected along the marine coast near Ulukhaktok, NT, in the western Canadian Arctic. This study provides critical insights into the ability of coastal Arctic Gadids to respond to shifts in resource use that are ongoing throughout their Arctic and sub‐Arctic distribution driven by climate change.

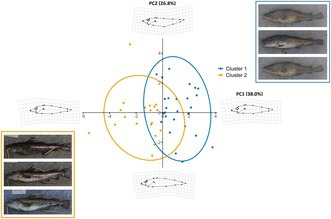

## 1 Introduction

Phenotypic variation among individuals of the same species (intraspecific variation) can result in conspecifics exhibiting contrasting ecological traits, with consequences for community structure and population‐level ecosystem function (Araújo et al. 2011; Des Roches et al. 2018; Ward et al. 2016). In wild animal populations, greater diversity in a given trait may stabilize populations against environmental disturbances (McKenzie et al. 2021; Nati et al. 2021) by buffering against their direct impact (Barabás and D'Andrea 2016; McKenzie et al. 2021) and enhancing subsequent population recovery (Des Roches et al. 2018). Consequently, it is increasingly recognized that wildlife conservation and management should aim to preserve or promote phenotypic diversity within wild populations (Des Roches et al. 2021; Moran et al. 2016; Ward et al. 2016) to mitigate anthropogenic impacts and maintain ecosystem resilience in an era of rapid environmental change. In fish, the underlying causes of intraspecific variation are complex and can be reflected in traits related to physiology, behavior, habitat use, and life history patterns (Burton et al. 2011; Metcalfe et al. 2016), but often correspond to adaptation to local or experienced conditions (Fraser et al. 2011). Of the phenotypic traits known to show significant variation within fish species, morphology is among the most widely studied and observed (Manna et al. 2019; Sampaio et al. 2013; Shuai et al. 2018; Webster et al. 2011).

Intraspecific variation in fish morphology is often associated with adaptations related to prey location, acquisition, and handling (Ferry‐Graham et al. 2002). For example, jaw and pharyngeal morphology was observed to be diet‐specific in a widespread cichlid fish species (Binning and Chapman 2010), while traits related to locomotory performance (e.g., body depth/length) and prey detection (e.g., eye diameter) have been found to correlate with individual dietary traits in European minnow ( *Phoxinus phoxinus* ) (Raffard et al. 2020). These morphological adaptations may also lead to individual fish specializing in the acquisition of certain prey items, such that their diet is dominated by specific prey types, which comprise a small proportion of those available to the overall population. As distributions of prey items are often associated with specific environmental variables, and the advantages provided by certain morphological traits may be environment‐dependent (Binning and Chapman 2010; Raffard et al. 2020; Svanbäck and Bolnick 2007), morphology may therefore also correlate with individual patterns of habitat use and selection (Paz Cardozo et al. 2021; Svanbäck and Bolnick 2007; Wolff et al. 2023). Individual variation in morphological traits may consequently drive intraspecific variation in both the “position” (i.e., the resource use of that individual) and “breadth” (i.e., the diversity of resources used by that individual, and their proportional importance) of their ecological niche (Paz Cardozo et al. 2021; Winkler et al. 2017).

The ecological niche can be further described through changes in diet and measured across various components, including differences in foraging areas (Mumby et al. 2018), prey consumption (Malek et al. 2016), generalization/specialization (Bond et al. 2016), and seasonal variability in diet (Coulter et al. 2019). These differences observed through isotopic variation can be measured from stable isotope ratios of tissues with different turnover rates and can reveal variation in these factors over short (days—weeks; plasma) to long time scales (weeks—months; red blood cells (RBCs); Vander Zanden et al. 2015) of sampled individuals within a population (Bearhop et al. 2004; Newsome et al. 2007). Carbon isotope ratios (^13^C/^12^C; *δ* ^13^C) are often used to investigate habitat use and movement patterns among individuals (Cherel and Hobson 2007; Matich et al. 2017) as they are representative of primary producers at the base of the food web, which undergo minimal fractionation during trophic transfer (∼0‰–2‰) (DeNiro and Epstein 1978). Nitrogen isotope ratios (^15^N/^14^N); (*δ* ^15^N) define an organism's trophic position, given notable enrichment in ^15^N with each trophic level (~2.3‰–5‰) (DeNiro and Epstein 1981; Post 2002). Variation in stable isotopes has been linked to distinct morphotypes to provide a complementary assessment of trophic and morphological variability (Senegal et al. 2021). Quantitative assessments of trophic variability can be measured through specialization indices (Bolnick et al. 2002), or further evaluated through linear mixed‐effects models (LMEs) to assess relative specialization within and between populations (Newsome et al. 2009).

Importantly, understanding how morphological trait diversity corresponds to an individual's position along the generalist‐specialist axis (Bolnick et al. 2002; Svanbäck and Schluter 2012) and the relative proportion of generalist or specialist phenotypes within a population can provide a more accurate indication of how natural populations will respond to environmental disturbances and their resilience to climate change (Hammond et al. 2018). Given that generalist individuals can make use of a broad range of resources, they may be less impacted by the loss of a given habitat or prey type, enhancing population stability (Laske et al. 2018). Where these generalists also exhibit a degree of resource partitioning (Chavarie et al. 2016), the impacts of environmental disturbances may be buffered further. In contrast, species with narrow resource needs are more likely to experience fitness consequences should a specific prey or habitat resource be lost, increasing their vulnerability to certain environmental stressors (Carscadden et al. 2020). However, specialized phenotypes can also enhance the potential for populations to expand into novel environments and niches (Martin and Pfennig 2009; Sexton et al. 2017), providing an alternative mechanism by which phenotypic diversity can contribute to population resilience.

Ongoing climate change impacts are transforming marine food web structure in Arctic ecosystems, changing the distribution, quality, and availability of resources that Arctic consumers depend on (Deb and Bailey 2023; Florko et al. 2021). Trends of poleward distribution shifts have been documented in marine species (Hastings et al. 2020), notably in boreal fish populations (Frainer et al. 2017; Kortsch et al. 2015). Sub‐Arctic Gadids such as Pacific cod ( *Gadus macrocephalus* ) and walleye pollock ( *Gadus chalcogrammus* ) have experienced significant shifts northward (Spies et al. 2020; Stafford et al. 2022), while endemic Arctic species including Arctic cod ( *Boreogadus saida* ) and saffron cod ( *Eleginus gracilis* ) are experiencing habitat contractions resulting in high potential for habitat overlap with northern invaders (Baker 2021; Laurel et al. 2016). The range expansion of these boreal generalists is expected to increase the rates of resource competition and predation experienced by endemic Arctic species (Bogstad et al. 2015; Fossheim et al. 2015), and represent stressors likely to have severe impacts on endemic Arctic Gadids (Geoffroy et al. 2023; Pettitt‐Wade et al. 2021). Additionally, sub‐Arctic Gadids have been shown to exhibit high adaptive potential to environmental stressors (Laurel et al. 2016; Leo et al. 2020), potentially exacerbating their competitive impacts on endemic Arctic Gadids as climate change continues.

Greenland cod ( *Gadus ogac* ) is endemic to the Arctic and northwest Atlantic Oceans and is broadly distributed throughout inshore coastal regions (McNicholl et al. 2017; Mikhail and Welch 1989). Subsistence cod fishing plays a role in Inuit tradition and livelihoods (Collings et al. 1998; Hoover et al. 2016; Pearce et al. 2011), with this species currently co‐managed by local, territorial, and government organizations (Lea et al. 2023). Changes in fish population dynamics in the western Canadian Arctic have been reported in recent years, including a decrease in abundance and an increase in size of Greenland cod (Chan S., Personal Communication, 24 July 2022). Additionally, communities in the Inuvialuit Settlement Region have reported changes in the marine ecosystem in recent years, including increasing water temperatures, rising water levels (Pearce et al. 2024, 2010), introduction of non‐native fish species (Chila et al. 2022), and an unusual sighting of a tunica bloom event (Pettitt‐Wade et al. 2020). The increasing prevalence of Pacific salmon has also been reported across the Inuvialuit Settlement Region, with many residents attributing this to ongoing climate change events (Chila et al. 2022; Lea et al. 2023; Pearce et al. 2024). Despite these changes, subsistence harvesting has increased over time (Lea et al. 2023), making them a focal research species given data deficiencies, community dependence, and year‐long presence in coastal areas. The limited information on Greenland cod behavior restricts our understanding of their interactions within the marine ecosystem and how they will respond to climate change.

To investigate the relationship between intraspecific trait diversity and generalist‐specialist behavior in Greenland cod in the context of a changing climate, we aimed to (i) quantify if morphological variation occurs in a population of Greenland cod using standard morphometric analysis, and (ii) estimate individual specialization‐generalization habitat‐trophic metrics for derived morphotypes using multi‐tissue stable isotope analyses. In the Arctic, where recent warming events and changes in the marine food web are being observed (Brandt et al. 2023), individual specialization may occur as a means to maximize fitness and reduce intraspecific competition among individuals (Roughgarden 1972).

## 2 Materials and Methods

### 2.1 Study Area and Fish Sampling

**FIGURE 1:**
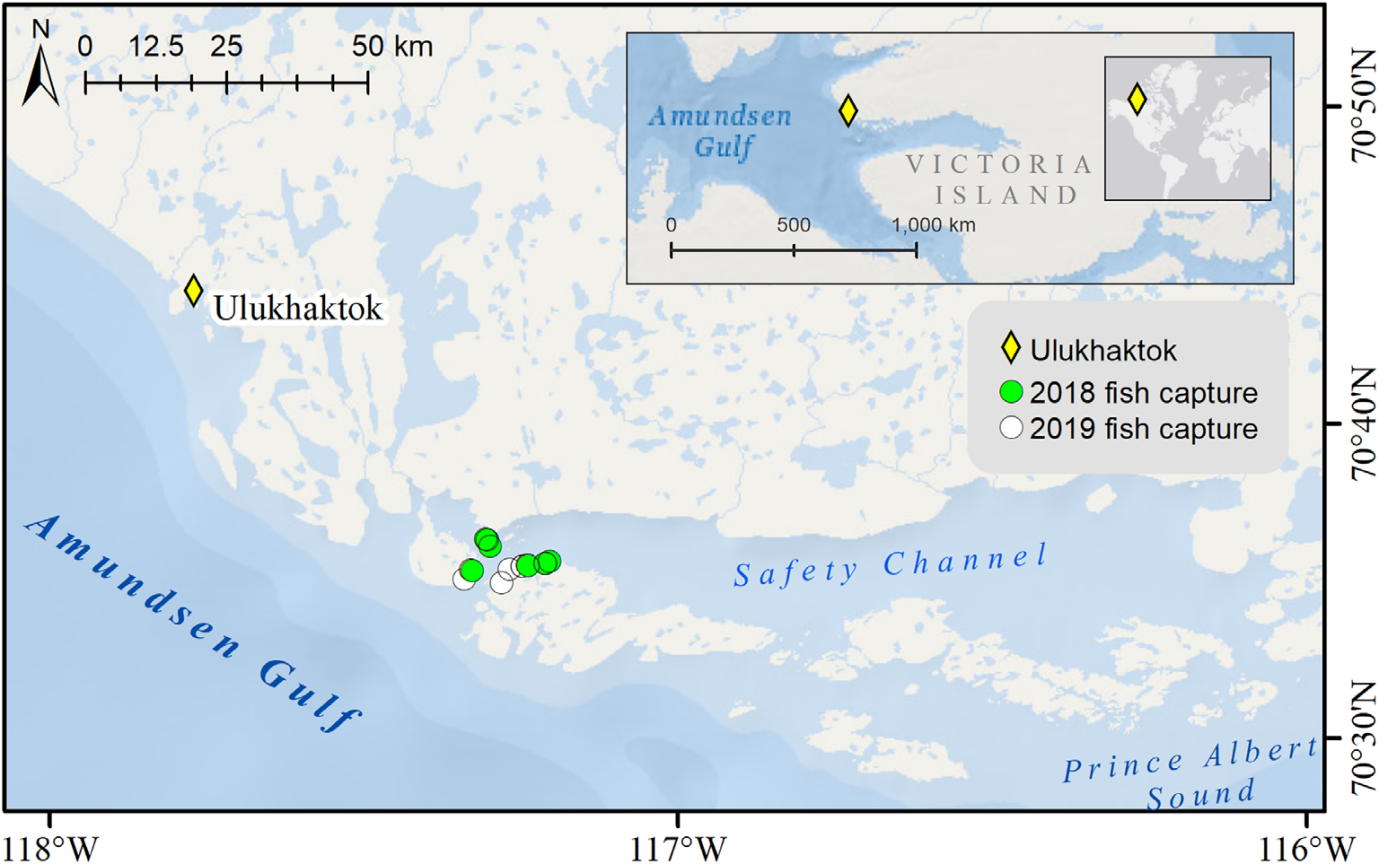
Map showing the study site in the western Canadian Arctic. Greenland cod ( *Gadus ogac* ) were captured in the semi‐enclosed Safety Channel near the community of Ulukhaktok, Northwest Territories (NT) in 2018 and 2019. Service layer credits: Esri, Garmin, GEBCO, NOAA.

Fish were captured near the Inuit community of Ulukhaktok, Northwest Territories (NT; 70.59° N, 117.27° E), at the entrance to Safety Channel, a semi‐enclosed channel approximately 30 km east of Ulukhaktok on the edge of the Amundsen Gulf in the western Canadian Arctic, Inuvialuit Settlement Region (Figure 1). The study location was determined following consultation and advice from the Olokhaktomiut Hunters and Trappers Committee (OHTC), Ulukhaktok Char Working Group (UCWG), and local Inuit harvesters (R. Klengenberg, I. Inuktalik, and D. Kuptana). Greenland cod were sampled from Safety Channel in 2018 during the summer months (July and August) and in 2019 during the spring and summer months (April, July–August). Fish were caught by angling with a rod and line from shore and from an 18‐ft vessel in open waters. After capture, individual Greenland cod were assigned a unique identification number and placed lateral side down on a mesh tray with a ruler and color chart. Fish were then photographed with a DSLR camera (Canon T2i Rebel) that was positioned above the fish on a fully extended tripod.

Following standardized photographs, blood was collected non‐lethally from the caudal vein of each individual using a 2 mL heparinized syringe and separated immediately into plasma and RBCs using a field centrifuge. Samples were stored frozen (−20°C) and later shipped to the University of Windsor, Canada for processing.

### 2.2 Geometric Morphometrics

#### 2.2.1 Data Preparation

**FIGURE 2:**
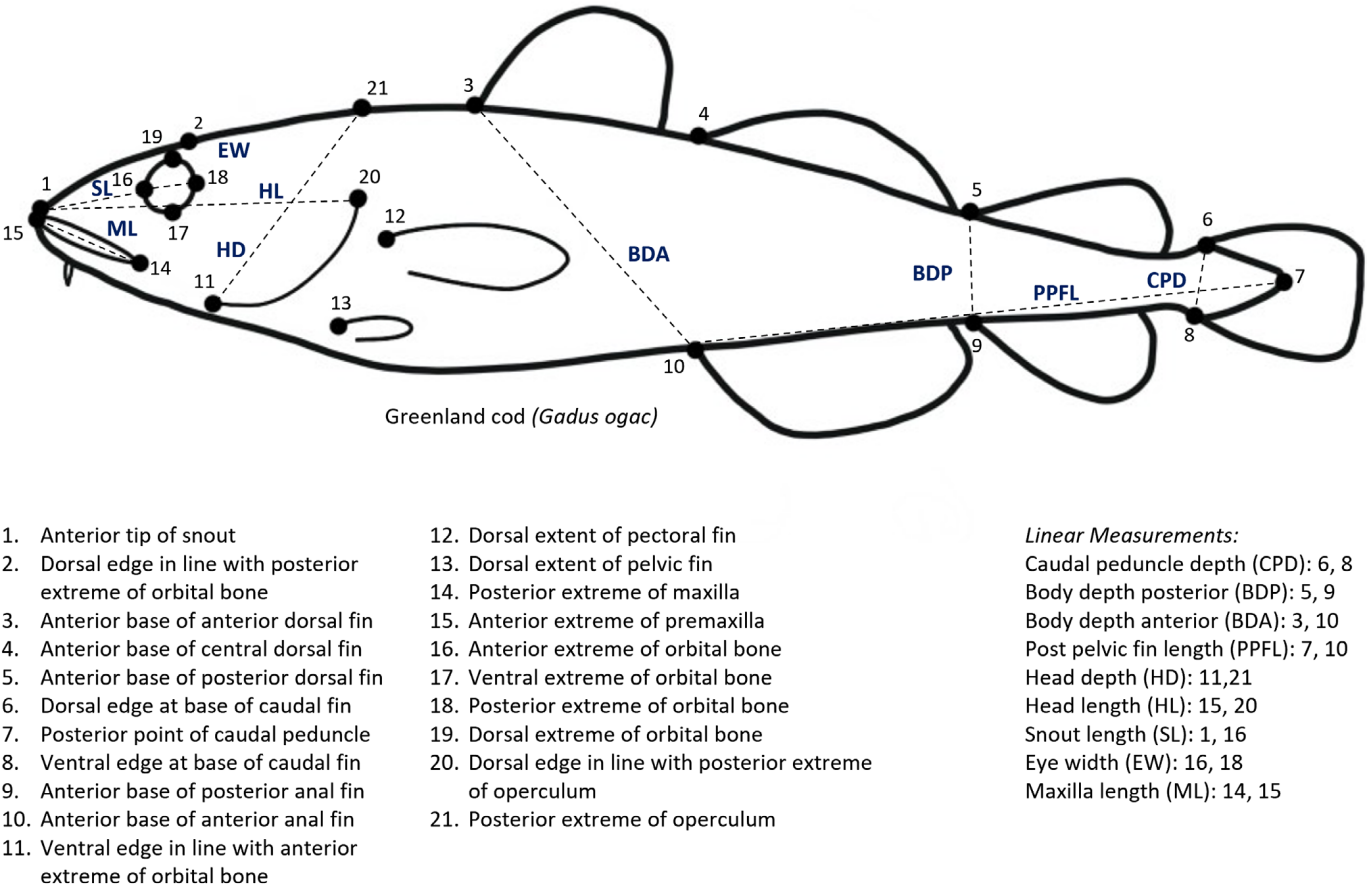
Locations of landmarks (*n* = 21) and linear measurements (*n* = 9) identified for geometric morphometric analysis of Greenland cod.

Morphometric analysis methods followed Skoglund et al. (2015) and Burke et al. (2022). Photographs of each fish were converted to a .tps file using the tpsUtil ver. 181 software (Rohlf 2021) and a total of 21 landmarks were identified to describe the head and body shape of the fish (Figure 2). Landmarks were selected according to previous morphometric studies examining the body shape of juvenile Atlantic cod (Marcil et al. 2006) and the head and body shape of Arctic char (Burke et al. 2022; Skoglund et al. 2015). Homologous landmarks were digitized on each photograph using the tpsDig2 ver. 2.31 software (Rohlf 2018). Photographs were scaled with a centimeter ruler before landmark placement. Fork length was measured (mm) as the distance between the tip of the snout and the posterior end of the caudal peduncle. For each photograph, individual landmarks were assigned a ranking of 1–3 for light and focus, following Burke et al. (2022): (1) representing poor quality, (2) average, or (3) excellent. An identical ranking system was then used to assess the overall quality of photographs; for example, if landmarks could not be easily distinguished on fish or excessive bending of a fish occurred, which would otherwise distort morphological analyses.

#### 2.2.2 Statistical Analyses

Landmark coordinates were standardized to remove the effects of size, position, and orientation on each image using the gpage() function within the R package geomorph ver. 4.0.1 (Adams et al. 2021) to obtain Procrustes coordinates. Principal component analysis (PCA) of Procrustes coordinates was performed using the gm.prcomp() function. Minimum and maximum eigenvalues were obtained to create deformation grids outlining the cod shape to visualize shape deviations relative to the mean position of each landmark using the plotRefToTarget() function.

Nine linear measurements were collected for each fish by calculating the distance between landmark pairs (Figure 2). Linear distances were size‐adjusted following Reist (1985):

$$\log^{10}Y_{i}=\log^{10}M_{i}+b\left(\log^{10}L_{m}-\log^{10}L_{i}\right)$$

where $Y_{i}$ is the size‐adjusted linear measurement, $M_{i}$ is the measured linear measurement, b is the linear regression coefficient (slope) of the measured linear measurement ($\log^{10}M_{i}$) against fork length ($\log^{10}L_{i}$), and $L_{m}$ is the average fork length across all fish and $L_{i}$ is the measured fork length. Linear measurements were allometrically aligned to the mean fork length of 36.81 cm.

PCA was applied to all nine size‐adjusted measurements using the PCA() function within the R package FactoMineR ver. 2.4 (Lê et al. 2008). The two principal components (PCs) explaining the most variation in the data were plotted on a two‐dimensional plane for data visualization. Using the silhouette method, the optimal number of clusters, *k*, was determined using the fviz_nbclust() function within the R package factoextra ver. 1.0.7 (Kassambara and Mundt 2020). PCA components were clustered into *k* groups using the kmeans() function within R (R Core Team 2020), such that the sum of squares from points to the assigned cluster centers is minimized. Two identified clusters were used to represent cod morphotypes within the sampled population.

A one‐way multivariate analysis of variance (MANOVA) was conducted to test for differences in the nine size‐adjusted linear measurements across clusters. Assumptions of normality (Shapiro–Wilk test) and equal variances (Levene's test) were conducted before MANOVA testing. MANOVAs with significant effects were followed up with *post hoc* Student *T*‐tests to determine which size‐adjusted linear measurements explained variation between clusters. To reduce the likelihood of Type I error, a Bonferroni correction was applied to the multiple comparisons.

### 2.3 Stable Isotope Analyses

#### 2.3.1 Data Preparation

Plasma and RBC samples were freeze‐dried and ground to a homogenous powder. Lipid extraction was conducted using the solvent distillation method. In brief, a 2:1 chloroform: methanol solution was added to the homogenized powder, agitated, and left in a 30°C water bath for 24 h. The solvent was then decanted, and samples were air‐dried using a fume hood. Samples and standards were then weighed into tin cups (5 mm × 9 mm) and analyzed using a 4010 Elemental Analyzer (Costech Instruments, Italy), coupled to a Delta Plus XL (Thermo‐Finnigan, Germany) continuous flow isotope ratio mass spectrometer (CFIRMS) at the University of Waterloo Environmental Isotope Laboratory. All resulting measurements were expressed in standard delta (*δ*) notation as parts per thousand differences (‰) relative to international standard reference materials for carbon (Vienna Pee Dee Belemnite; Coplen et al. 2002) and nitrogen (atmospheric nitrogen; IAEA 1995), using the following equation:

$$\mathrm{\delta R}\;\left(‰\right)=\left[R_{\text{sample}}/R_{\text{standard}}–1\right]\times 1000$$

where *R* is the ratio of ^13^C/^12^C or ^15^N/^14^N. Analytical precision was ±0.2‰ and ±0.3‰ for *δ* ^13^C and *δ* ^15^N respectively, where reference materials of USGS 40 and USGS 41 from L‐glutamic acid were run in duplicates after every ten samples.

#### 2.3.2 Statistical Analyses

To derive information on temporal habitat‐trophic shifts at the individual level, analyses of stable isotopes (^13^C and ^15^N) were performed on multiple tissues per individual (RBC and plasma, i.e., slow vs. fast turnover). Comparison of different turnover rates (RBC vs. plasma) provides an indication of habitat‐trophic switches over time, with RBC representing weeks to months and plasma representing days to weeks of diet history (Vander Zanden et al. 2015). Variability in tissue lipid content can often impact bulk isotope values; therefore, lipid extraction was done on blood samples to maintain the reliability of individual estimates, particularly in *δ* ^13^C (Post et al. 2007). Raw isotope values were then corrected with diet‐tissue discrimination factors to standardize values given known tissue‐specific isotopic discrimination. Inclusion of discrimination factors accounted for discrepancies between an individual and their diet (Δ^13^C = *δ* ^13^C_consumer_ − *δ* ^13^C_food_ and Δ^15^N = *δ* ^15^N_consumer_ − *δ* ^15^N_food_), thus providing a more accurate representation of ^13^C and ^15^N values for that individual. Diet‐tissue discrimination factors based on a controlled study of the leopard coral grouper ( *Plectropomus leopardus* ) were applied (*δ* ^13^C: plasma; 1.2‰, RBC; 0.1‰ and *δ* ^15^N: plasma; 0.9‰, RBC; 1.1‰) (Matley et al. 2016). Given the absence of species‐specific discrimination factors for Greenland cod, these values were selected based on comparable tissue types (Dalerum and Angerbjörn 2005), lipid extraction treatment (Murry et al. 2006), environment type (marine; Vanderklift and Ponsard 2003), and biology (Frisch et al. 2016; Mikhail and Welch 1989). Discrimination‐corrected RBC and plasma values were first compared between morphotype cluster groups using Student's *T*‐tests for *δ* ^13^C and *δ* ^15^N separately. Assumptions of normality (Shapiro–Wilk test) and equal variances (Levene's test) were examined prior to analysis.

#### 2.3.3 Habitat‐Trophic Metrics

Habitat‐trophic specialization was determined using LMEs on individual *δ* ^13^C and *δ* ^15^N values for the morphological cluster groups and the total sample population. Following Newsome et al. (2009), separate LMEs for *δ* ^13^C and *δ* ^15^N were used to analyze isotopic variance using the lmer() function within the R package lme4 ver. 1.1‐27.1 (Bates et al. 2015); fork length and tissue type were assigned as fixed effects, and individual fish ID as a random effect for the full model. Variation displayed in *δ* ^13^C and *δ* ^15^N collected in RBC and plasma tissues from individual cod could be explained by a combination of the fixed and random effects. The residual variance corresponds to the within‐individual component (WIC), where smaller residual values represent fewer diet/trophic switches and greater residual values represent more diet/trophic switches. Variance between individual fish represents the between‐individual component (BIC). The sum of BIC and WIC represents the total niche width (TNW) of the population. Diet specialists are broadly defined as having a narrow niche (WIC) relative to the total niche (TNW) (Bates et al. 2015; Bolnick et al. 2003), while diet generalists display greater WIC values as a result of higher residual variance. Individual specialization (IS) metrics were calculated and standardized as the proportion of WIC:TNW for comparison between morphological cluster groups.

#### 2.3.4 Individual Specialization Metrics

To calculate individual WIC values, separate *δ* ^13^C and *δ* ^15^N predicted linear models were fit to RBC versus plasma isotope values. The absolute difference between the predicted and actual values represents residual variance and was thus used to estimate individual WIC values. The effects of cluster, fork length, and sample year on individual WIC values for *δ* ^13^C and *δ* ^15^N were examined via general linear models (GLMs) using the glm() function in R (R Core Team 2020). Non‐significant interaction terms were dropped sequentially, starting with those with the smallest *t* values, but were retained if their removal resulted in higher AIC values [ΔAIC > 2 (Arnold 2010)]. Assumptions of normality and multicollinearity of the response variable were checked, and WIC values were log10 transformed prior to testing to meet the assumptions. All statistical analyses were performed using R software, Ver. 4.1.1 (R Core Team 2020). Significance was tested at α = 0.05.

## 3 Results

### 3.1 Morphological Variation and Morphotypes

A total of 117 Greenland cod (2018: *n* = 69, 2019: *n* = 48) ranging in size from 251.9 mm to 578 mm (mean 388.58 ± 57.63 mm SD, *n* = 102 where length was available) and weight from 250 g to 2000 g (mean 882.04 ± 340.93 g SD, *n* = 109 where weight was available) were caught and sampled for geometric morphometric analyses. Of those, 72 were excluded following our assessment criteria, resulting in a final sample size of 45 fish (2018: *n* = 19, 2019: *n* = 26).

**FIGURE 3:**
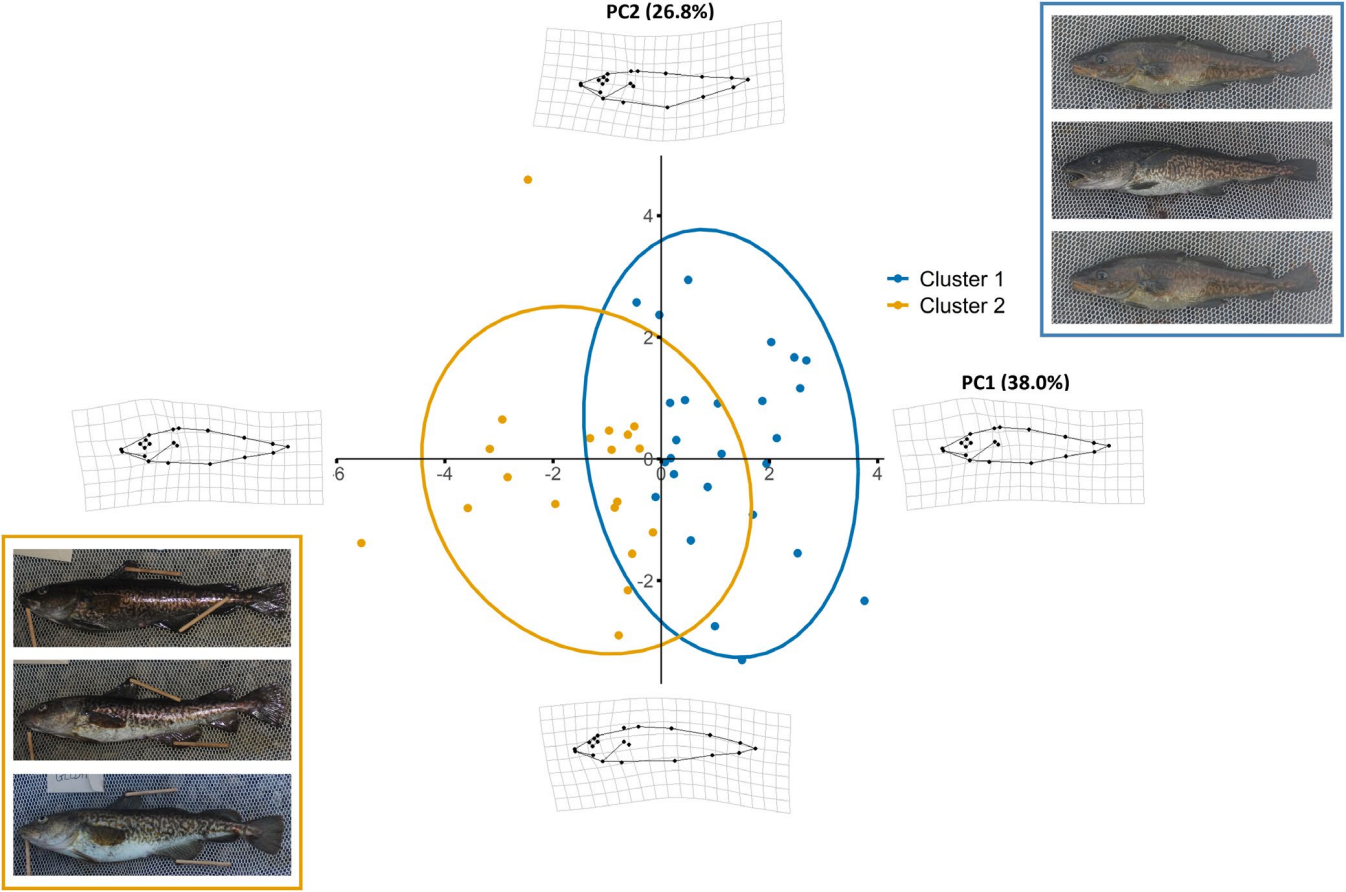
Principal component analysis (PC1 and PC2) of body shape for the two identified morphotype cluster groups of Greenland cod using kmeans clustering. Deformation grids represent shape variation along each extreme of the axes (PC1 on *X* axis, PC2 on *Y* axis). PC1 accounted for the greatest variation in head features (head length, head depth, snout length, eye width) and PC2 accounted for the greatest variation in posterior features (body depth posterior and caudal peduncle depth). Example photographs are shown for each morphotype cluster group. Cluster 1 (blue) is defined by a large head and stocky body, whereas cluster 2 (yellow) is defined by a small head and slender body.

**FIGURE A1:**
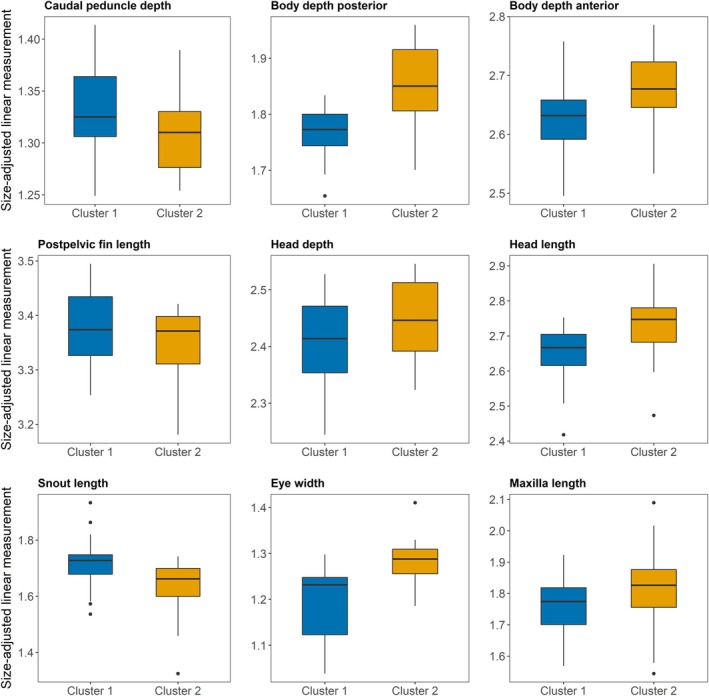
Box plot comparisons of nine size‐adjusted linear measurements for identified morphotype cluster groups of Greenland cod (cluster 1: *N* = 26, cluster 2: *N* = 19). Boxplots identify the median (black line), the first and third quartiles (top and bottom of box), and whiskers (vertical lines) showing the range in linear measurements.

For the PCA conducted on the nine size‐adjusted linear measurements, PC1 and PC2 accounted for 64.7% of the total variation (Table A1). PC1 accounted for 38% of the total variation, principally from variation in head features, including head length, head depth, snout length, and eye width (Figure 3). Through deformation grids, extreme positive PC1 values showed more deformations in the body, resulting in a longer head depth and enlarged body depth anterior (cluster 1). Extreme negative PC1 values showed deformations centered around the intersection of the head and body regions, resulting in a shortened head and reduced body depth anterior (cluster 2). PC2 accounted for 26.8% of the total variation and was explained mainly by body depth posterior, and caudal peduncle depth. Variation in PC2 values was mainly present in the posterior end of the fish. However, the natural bending of the fish during photography may have also contributed to these deformations. Subsequent clustering analysis of PC1 and PC2 data revealed two morphotype clusters, with head features explaining the majority of this distinction (Figures 3 and A1). Morphotype cluster 1 was found to have a larger head and stockier body, whereas morphotype cluster 2 was found to have a smaller head and slender body.

MANOVA indicated that linear measurements differed significantly between cluster groups (Pillai's Trace: *F* = 6.20, *p* < 0.001; Table 1). *Post hoc* Student *T*‐tests showed significant differences between morphotype cluster groups for all size‐adjusted linear measurements with the exception of the caudal peduncle (*t* = −1.3, *p* = 0.19). This was despite the fact that the caudal peduncle contributed the most to the variation observed in PC2. Post pelvic fin length (PPFL) had the strongest overall effect on the difference between the clusters (*t* = 25.9, *p* < 0.001), while of all head features, head depth, head length, snout length, eye width, and maxilla length significantly differed between the two clusters. Of the head features, head length and head depth contributed the most variation (*t* = 16.8, *p* < 0.001 and *t* = 13.4, *p* < 0.001, respectively).

**TABLE 1:** Summary of size‐adjusted linear measurements for Greenland cod (mean ± standard error (SE) and range in mm).

Linear measurement		Sample population mean ± SE, range (mm)	Morphotype mean measurement (mm)		*T*‐test		
			Cluster 1	Cluster 2	*t*	df	*p*
CP	Caudal peduncle	1.32 ± 0.001, 1.25 to 1.41	1.33	1.31	−1.3	44.7	0.19
BDP	Body depth posterior	1.82 ± 0.01 1.65 to 1.96	1.84	1.78	5.2	46	< 0.001
BDA	Body depth anterior	2.66 ± 0.001 2.50 to 2.79	2.69	2.62	16.5	45.5	< 0.001
PPFL	Postpelvic fin length	3.37 ± 0.01 3.18 to 3.49	3.38	3.35	25.9	45.6	< 0.001
HD	Head depth	2.43 ± 0.01 2.25 to 2.55	2.47	2.38	13.4	45.9	< 0.001
HL	Head length	2.69 ± 0.01 2.42 to 2.91	2.74	2.62	16.8	47.2	< 0.001
SL	Snout length	1.68 ± 0.02 1.32 to 1.93	1.72	1.63	3.4	47.6	< 0.05
EW	Eye width	1.25 ± 0.01 1.04 to 1.41	1.28	1.20	−2.3	45.9	< 0.05
ML	Maxilla length	1.79 ± 0.02 1.54 to 2.09	1.83	1.74	4.9	48.5	< 0.001

*Note:* Post hoc Student *T*‐tests show comparisons between morphotype cluster 1 (*n* = 26) and cluster 2 (*n* = 19) for each linear measurement (mean in mm).

### 3.2 Habitat‐Trophic Metrics

**FIGURE 4:**
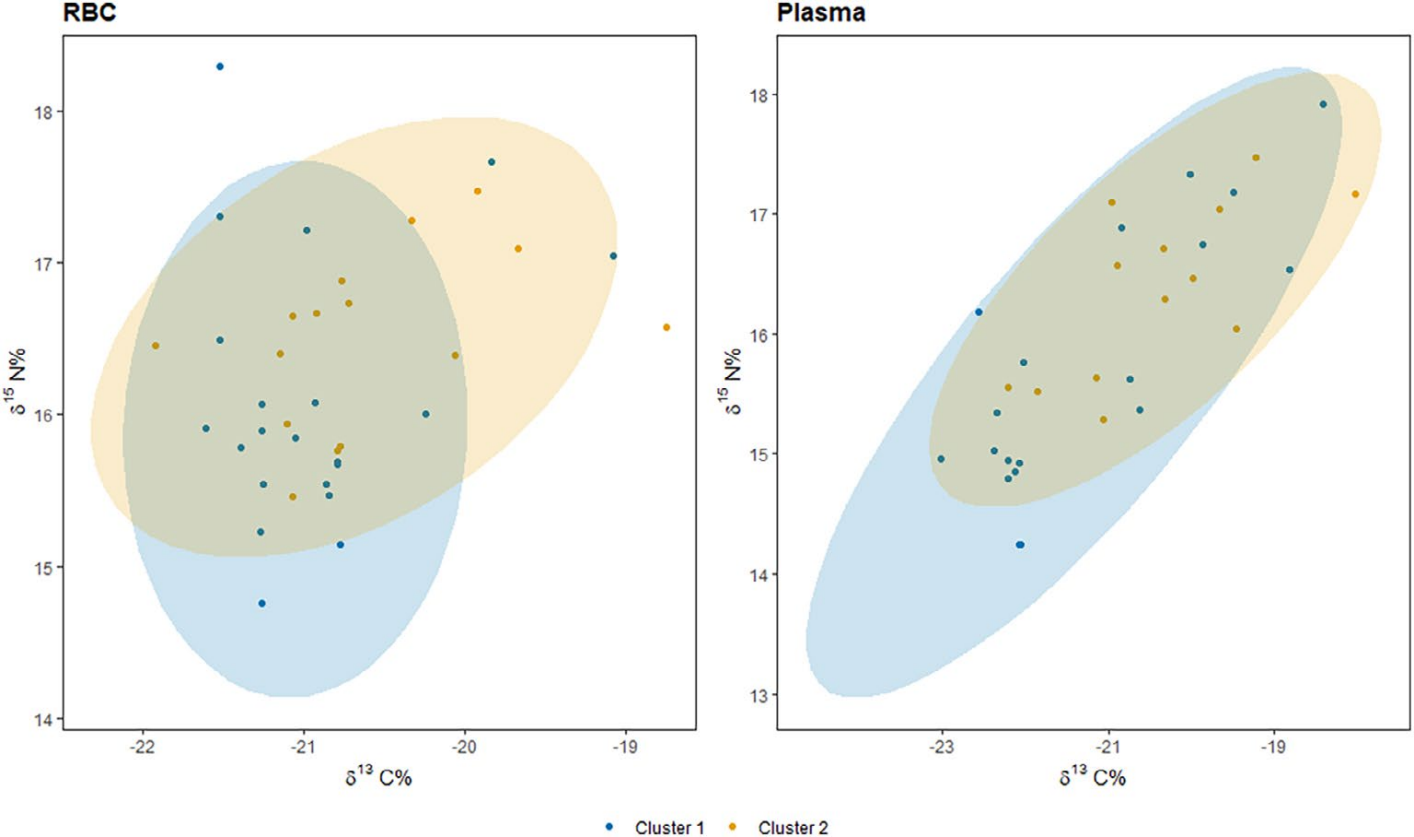
Biplot of *δ* ^13^C and *δ* ^15^N for RBC (left) and plasma (right) tissue of individual Greenland cod separated by morphotype cluster groups 1 and 2. Ellipses are illustrated with 95% confidence.

A total of 45 RBC and plasma samples (cluster 1: *n* = 26, cluster 2: *n* = 19) were analyzed for *δ* ^13^C and *δ* ^15^N (Table 2, Figure 4). Discrimination‐corrected *δ* ^13^C values ranged from −22.02‰ to −18.85‰ in RBC and from −24.22‰ to −19.23‰ in plasma. While RBC and plasma *δ* ^13^C values were slightly higher in cluster 2 compared to cluster 1, no significant difference between cluster groups for RBC (*t* = −1.5, df = 26, *p* = 0.15) or plasma (*t* = −2.0, df = 26, *p* = 0.06) was observed. Discrimination‐corrected *δ* ^15^N values ranged from 13.66‰ to 17.19‰ in RBC and from 13.35‰ to 17.01‰ in plasma. No significant differences in the mean were observed between cluster groups for both RBC (*t* = −1.5, df = 34, *p* = 0.13) or plasma (*t* = −1.8, df = 29, *p* = 0.08).

**TABLE 2:** Summary of discrimination‐corrected ^13^C and ^15^N values (mean ± standard error (SE) and range in ‰) of RBC and plasma for identified morphotype cluster groups of Greenland cod.

		Cluster 1		Cluster 2		*T*‐test		
	Tissue	*n*	Mean ± SE, range (‰)	*n*	Mean ± SE, range (‰)	*t*	df	*p*
^13^C	RBC	26	−21.05 ± 0.13 −21.71 to −19.18	19	−20.7 ± 0.20 −22.02 to −18.85	−1.5	26	0.15
	Plasma	26	−22.46 ± 0.32 −24.22 to −19.61	19	−21.59 ± 0.32 −23.41 to −19.23	−2.0	26	0.06
	*δ* ^13^C (RBC – plasma)	19	1.42 ± 0.32 −2.01 to 2.88	12	0.86 ± 0.22 −0.16 to 2.19	1.5	28	0.15
^15^N	RBC	26	15.03 ± 0.20 13.66 to 17.19	19	15.40 ± 0.15 14.36 to 16.37	−1.5	34	0.13
	Plasma	26	14.83 ± 0.25 13.35 to 17.01	19	15.47 ± 0.20 14.39 to 16.57	−1.8	29	0.08
	*δ* ^15^N (RBC – plasma)	19	0.22 ± 0.16 −1.71 to 1.63	12	−0.12 ± 0.20 −1.45 to 1.16	1.3	23	0.19

*Note:* Results from Student *T*‐tests comparing morphotype clusters are also shown.

**FIGURE 5:**
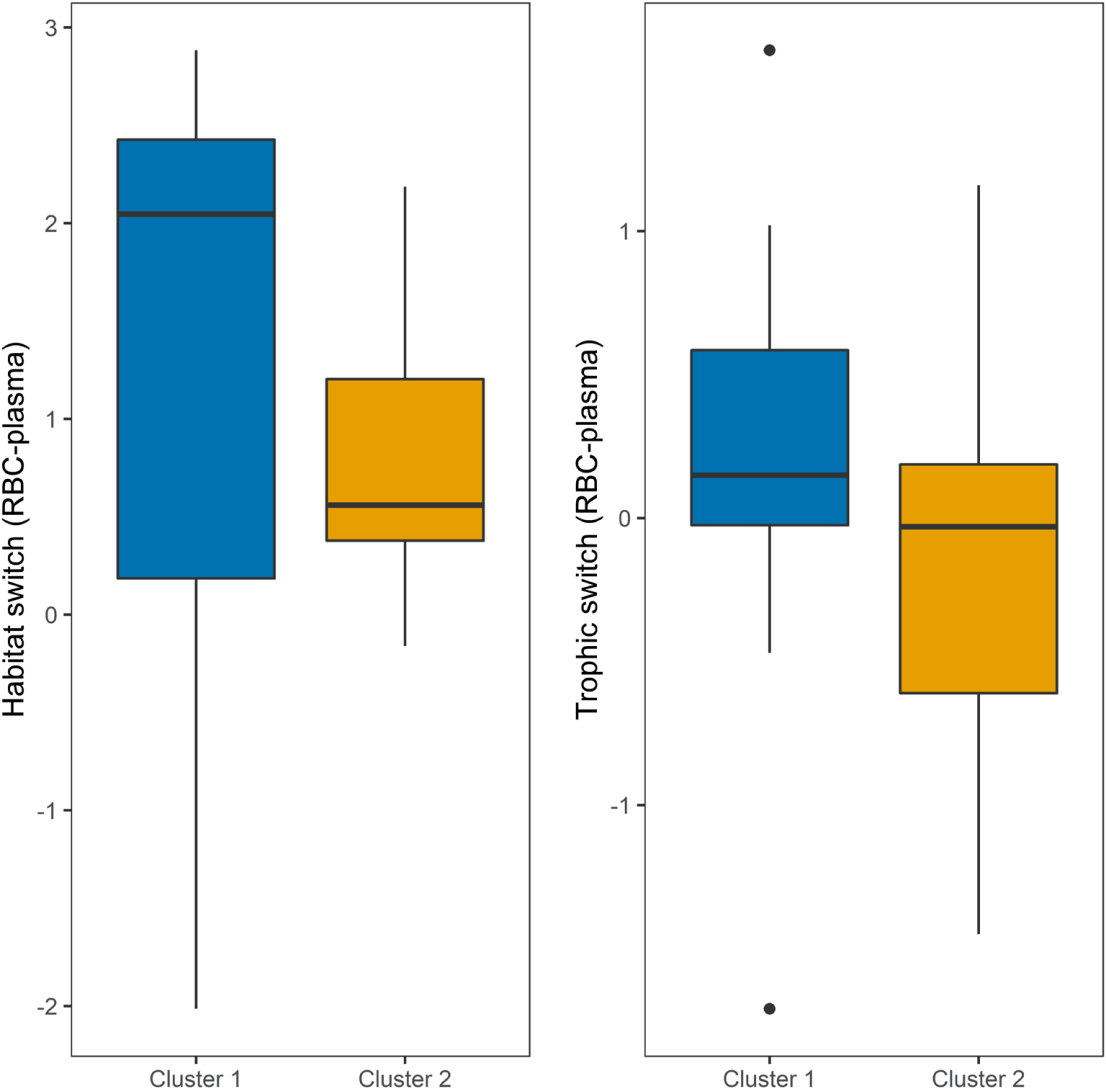
Comparison of discrimination‐corrected habitat (left; *δ* ^13^C) and trophic (right; *δ* ^15^N) switch metrics (RBC‐plasma) between the two identified morphotype cluster groups, with no significant difference between cluster groups. Boxplots identify the median (black line), the first and third quartiles (top and bottom of box), and whiskers (vertical lines) showing the range in habitat and trophic switch values.

To examine habitat and trophic shifts (RBC—plasma; *δ* ^13^C and *δ* ^15^N), individual fish that were missing either RBC or plasma values were removed, providing a total of 31 individuals with paired tissue isotope data (cluster 1: *n* = 19, cluster 2: *n* = 12; Figure 5, Table 2). Student *T*‐tests revealed no significant difference between cluster groups for either *δ* ^13^C (*t* = 1.5, df = 28, *p* = 0.15) or *δ* ^15^N (*t* = 1.3, df = 23, *p* = 0.19).

### 3.3 Individual Specialization Metrics

**FIGURE 6:**
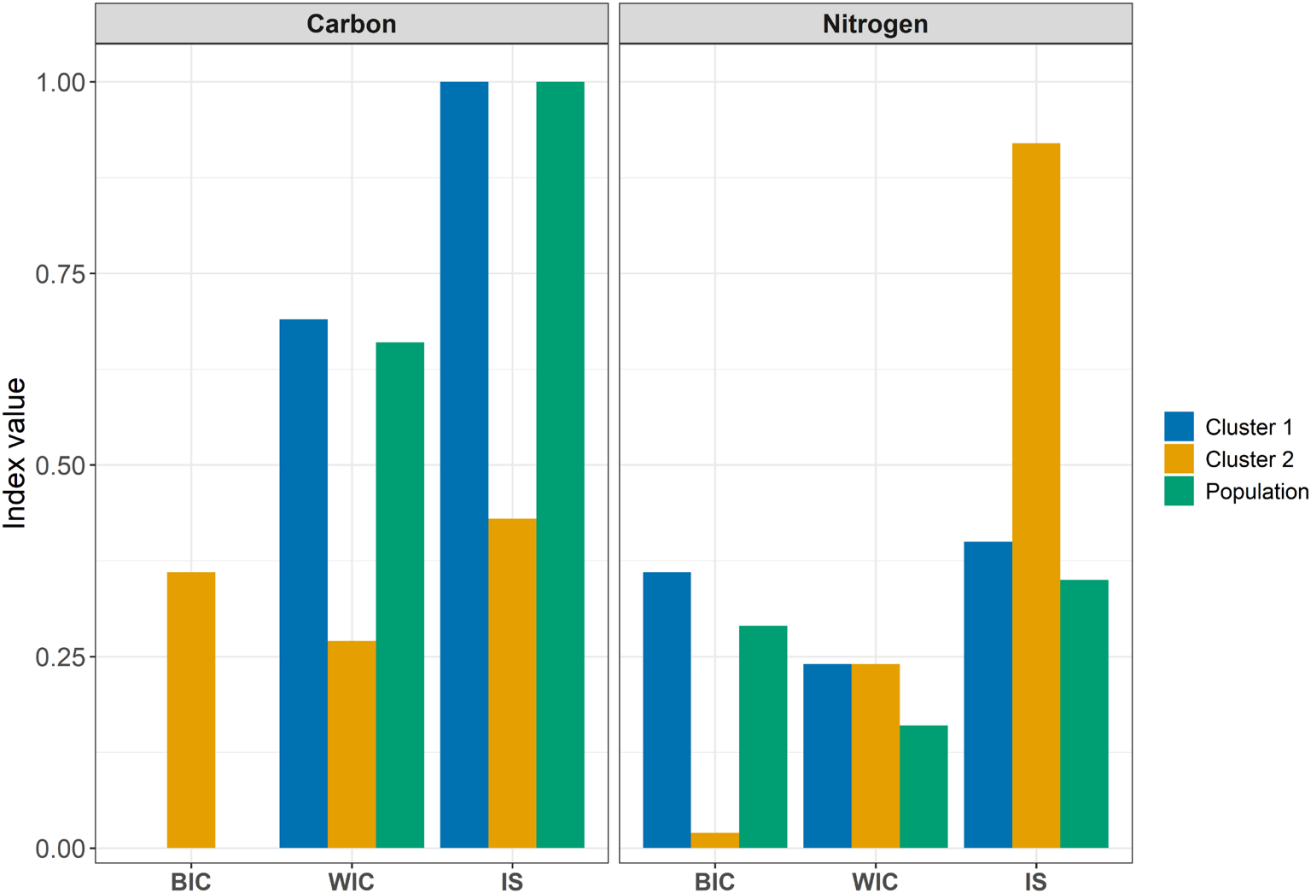
Individual specialization indices derived from discrimination‐corrected lipid‐extracted *δ* ^13^C (Carbon; left panel) and *δ* ^15^N (Nitrogen; right panel) stable isotope values for morphotype cluster 1, cluster 2, and total sample population of Greenland cod derived from linear mixed‐effects models. Between individual component (BIC), within individual component (WIC) and individual specialization (IS) indices are shown.

The *δ* ^13^C LME revealed that cluster 1 (large head and stocky body) and population displayed lower habitat specialization compared to cluster 2 (small head and slender body) (WIC:TNW: 1.00 (cluster 1); 0.43 (cluster 2); 1.00 (population), Figure 6). BIC for cluster 2 was higher (0.36) in comparison to cluster 1 (0.00) and population (0.00). WIC was highest in cluster 1 (0.69) and population (0.66), and lowest in cluster 2 (0.27). The *δ* ^15^N LME revealed that cluster 2 displayed lower trophic specialization compared to cluster 1 and total population (WIC:TNW: 0.40 (cluster 1); 0.92 (cluster 2); 0.35 (population), Figure 6). BIC for cluster 1 was higher (0.36) in comparison to population (0.29) and cluster 2 (0.02). WIC was higher in both clusters 1 (0.24) and 2 (0.24), compared to the population (0.16).

### 3.4 Factors Affecting Individual Specialization

Individual *δ* ^13^C WIC values of cod ranged from 0.035 to 3.15 (mean 1.05 ± 0.57 SD), with 55% of the total sample population having an index value < 0.5. WIC values for *δ* ^13^C showed a significant positive relationship with fork length and were significantly higher for fish in morphotype cluster 1 and those sampled in 2019 (*p* > 0.05; Table 3). Individual *δ* ^15^N WIC values ranged from 0.013 to 1.72 (mean 0.50 ± 0.47 SD), with only 14% of the total sample population having a WIC value < 0.5. WIC values for *δ* ^15^N were not associated with cluster, fork length, or sample year (*p* > 0.05; Table 3).

**TABLE 3:** General linear model results for the effects of morphotype cluster, fork length, and sample year on *δ* ^13^C and *δ* ^15^N within individual component (WIC) values for individual Greenland cod.

Model	Parameter	Estimate	SE	*p*
^13^C WIC	Cluster	0.49	1.31	0.01
	Fork length	1.00	1.00	0.003
	Year	1.92	1.34	0.03
^15^N WIC	Cluster	1.40	1.79	0.57
	Fork length	1.00	1.00	0.48
	Year	1.24	1.88	0.73

*Note:* Note that estimates and standard errors (SE) were back‐transformed from their common logarithm (log_10_). Comparison of AIC results in the full model being used for both ^13^C WIC and ^15^N WIC.

## 4 Discussion

In the current study, we quantified the extent that morphotype correlated with individual specialization‐generalization using habitat‐trophic metrics in Greenland cod. We identified two morphotypes of Greenland cod that differed primarily in head length and body depth. Mean stable isotope values of *δ* ^13^C and *δ* ^15^N from blood tissues showed minimal differences between morphotypes, but measures of IS indicated that the morphotype with the smaller head and slender body had lower habitat specialization and higher trophic specialization compared to the morphotype with the larger head and stockier body. Importantly, there was high variation in habitat‐trophic metrics across individuals for both morphotypes. We postulate that the morphotypes linked to high variation in habitat‐trophic metrics can display a gradient of generalist‐specialist traits, rather than distinct morphotypes assigned to either generalists or specialists. Our findings informed on the ecological consequences of morphological variation in marine fish and furthered our understanding of the potential response to shifts in habitat and trophic resources that are occurring as a result of a changing climate.

Intraspecific variation in morphological traits was observed in the sampled Greenland cod population, which can arise from individual differences in resource acquisition (Binning and Chapman 2010) and habitat use (Winkler et al. 2017). The larger head paired with a stockier body associated with morphotype cluster 1 may be beneficial for consuming multiple or large‐sized prey items and specialized for low‐speed maneuvering and navigating complex habitats with precision, such as rocky benthic areas (Segura et al. 2015; Webb 1984). Gape size can dictate prey selection, with larger‐sized heads selecting larger prey items (Mihalitsis and Bellwood 2017). Contrastingly, the smaller head and slender body associated with morphotype cluster 2 are better suited for consuming fewer (i.e., in one event) or smaller prey items, with a more streamlined body allowing for greater speed and improved maneuverability (Webb 1984). These observed differences in morphological specializations could represent performance trade‐offs based on handling efficiency linked to gape size and encounter rates according to maneuverability and habitat use. With the anticipated impacts of climate change, performance trade‐offs associated with the different morphotypes could drive further diversification between these groups. The minimal distinctions between these morphotype groups may become more apparent in the future, resulting in the exploitation of different resources and minimal competition between groups. In addition to selection pressures related to life history and behavior patterns, some morphotypes may be more vulnerable to other pressures, such as fisheries‐induced selection (Alós et al. 2014; Hollins et al. 2018). As seen in morphotype cluster 1, these larger‐mouth individuals may be more vulnerable to hook‐and‐line methods (Alós et al. 2014) while gill nets can easily retain these larger‐bodied fish (Reis and Pawson 1999), both of which are frequent methods of catch used by the community. As a result, the Greenland cod fishery in Ulukhaktok may be impacted by fishing habits that can selectively remove particular phenotypes.

Performance trade‐offs linked to morphology can be observed across populations occupying overlapping niches. It was proposed that three distinct morphotypes of migratory anadromous Arctic char ( *Salvelinus alpinus* ) that were also sampled in Ulukhaktok, NT, and Safety Channel were likely driven by ecological differences linked to resource or habitat use (Burke et al. 2022). Anadromous fish such as Arctic char may be more likely to diverge into multiple morphotypes than Greenland cod due to contrasting life history patterns, where migratory Arctic char encounter marine, estuarine, or freshwater environments (Hollins et al. 2022; Moore et al. 2016), and can spend proportionally more time in one environment versus another (e.g., long vs. short river runs; long vs. short sea‐run migrations to core foraging habitat). This could promote diversification into different morphotypes as a result of contrasting phenotypes optimized to occupy a diverse array of environments, encouraging the potential for niche specialization (Hollins et al. 2022). In contrast, non‐migratory Greenland cod inhabit marine environments year‐round, which may help explain the reduced phenotypic diversity or morphotypes required to adapt to the environments they occupy. It is apparent, however, that the morphotypes of both species can be displayed over a gradient rather than as distinct morphotypes.

Our findings from individual‐linked morphological and stable isotope data demonstrate that Greenland cod display variation in feeding behaviors across morphotypes. The morphotype with the elongated head and stockier body (cluster 1) displayed higher habitat specialization (i.e., restricted habitat range or consumption of basal carbon sources; *δ* ^13^C values) and lower trophic specialization (i.e., feeding across trophic levels; *δ* ^15^N values). The stockier body associated with morphotype cluster 1 could reflect reduced maneuverability and speed (Webb 1984), leading to generalist foraging behaviors within a limited range of habitats. Contrastingly, the morphotype with the shorter head and slender body (cluster 2) displayed lower habitat specialization (i.e., occupied a broader foraging habitat or consumed more diverse basal carbon) and higher trophic specialization (i.e., feeding on prey at a similar trophic level). The slender body could be more advantageous for foraging activities at higher speeds (Webb 1984) and allow individuals to be selective, or specialize on particular prey. Patterns of functional morphology have been correlated with feeding behaviors in a variety of fish species, where benthivorous feeding has been documented in deeper‐bodied fish (cluster 1) and limnetic feeding activities have been linked to shallower‐bodied fish (cluster 2; Robinson and Parsons 2002).

The variations we observed in habitat‐trophic metrics and non‐significant differences in bulk tissue *δ* ^13^C and *δ* ^15^N between morphotypes could reflect a gradient of habitat and/or trophic segregation among coastal versus offshore areas and benthic versus pelagic feeding (Hobson et al. 1995), respectively. The fact that isotopic distinction was marginal across the two morphotypes could suggest that morphology is not a key factor regulating feeding in a specific habitat. Alternatively, morphology may allow greater flexibility whereby individual fish do not undertake distinct movement behaviors, but spend proportionally more time feeding in certain habitats compared to others. The range in individual WIC values and absolute stable isotope tissue differences we observed across the two morphotypes supports the hypothesis that cod are adapted to high connectivity across habitat types. The inclusion of spatial movement data of individual fish and baseline isotopic data of the marine environment (Rogers and White 2007) could help further tease apart the causation of morphological differences and observed specialization values and ranges.

While high variation in *δ* ^13^C and *δ* ^15^N values was observed within clusters, it may be insufficient for individuals to diverge into discrete morphotypes with entirely distinct patterns in resource use, suggesting evidence of a population‐level generalist niche. Given that cod were sampled in a relatively small area (~5 km^2^) characterized as a coastal benthic environment, this area may support two morphotypes by providing a relatively consistent pool of resources suitable for cod that aligns with a generalist feeding strategy. However, intraspecific variation in niche use (i.e., isotopic niche plasticity, in both habitat and prey consumed) could occur within the population to alleviate selection pressures and reduce competition among individuals (Pettitt‐Wade et al. 2015; Skulason and Smith 1995). Greenland cod consumes small fish, crustaceans, and mollusks (McNicholl et al. 2017) and are found in structurally complex areas (Knickle and Rose 2014). Arctic coastal habitats are also considered highly dynamic and biologically complex (Irrgang et al. 2022), which creates opportunities for fish to occupy such environments year‐round for feeding and habitat use (Friedman et al. 2020; Kutti et al. 2015), potentially allowing individuals with a wide range of traits to inhabit and thrive in these mixed environments.

Annual ice breakup can greatly influence the availability of resources in the Arctic, leading to seasonal resource pulses (Yang 2009). In our study, sampling of Greenland cod occurred during the ice‐free season following a resource pulse event, where the observed habitat‐trophic switches could represent a period of higher activity due to increased productivity and prey availability following ice melt (Hermann et al. 2023; Hop et al. 2011). Seasonal prey shifts documented from stomach content analysis revealed that Greenland cod in eastern James Bay shift from higher trophic level feeding in the winter to lower trophic feeding in the summer, likely due to temperature and prey availability (Morin et al. 1991). Therefore, the longer turnover rate of RBC (weeks—months) in our study may have captured winter pre‐ice breakup conditions and could reflect feeding on higher trophic level prey during that time for our sample population. The non‐significant difference between morphotype clusters in habitat and trophic switch values (absolute RBC‐plasma) seems to suggest that resource pulses could have little to no impact on feeding activity over time and may be similar for both morphotype groups. Indeed, other literature supports the concept of year‐round feeding on similar resources (Mikhail and Welch 1989; Morin et al. 1991) and why this may promote generalist feeding behavior (Pettitt‐Wade et al. 2023). However, the high degree of variation in specialist and generalist behaviors indicated by our LMEs suggest that some individuals are responding differently to environmental fluctuations (e.g., resource pulse or local upwelling).

Intraspecific differences in habitat‐trophic variation derived from *δ* ^13^C and *δ* ^15^N GLMs at the individual level showed morphotype cluster, fork length, and sample year had significant effects on *δ* ^13^C WIC values but no effect on *δ* ^15^N WIC values. This indicates that habitat segregation (from *δ* ^13^C values) is the factor most discriminating the two morphotype clusters, most likely tied to differences in head and body shape. This is supported by previous research that found the direction of habitat shifts (*δ* ^13^C tissue difference) in Greenland cod changed with body size (Pettitt‐Wade et al. 2023). The lack of association between morphotype cluster and trophic level in the current study may be due to the overlapping trophic use of prey resources between morphotypes. Ontogenetic patterns in *δ* ^15^N variation among tissues showed that Greenland cod were trophic generalists regardless of body size (Pettitt‐Wade et al. 2023). This is in agreement with the limited effect of size in our *δ* ^15^N individual WIC GLM. Fish with larger mouths have the ability to ingest prey of various sizes (i.e., less gape limited) and may result in a wider trophic niche (Jennings et al. 2002; Layman et al. 2005). This pattern may be observed with the larger head and body associated with morphotype cluster 1; however, trophic shifts (*δ* ^15^N) did not correlate with body length for these same individuals (Pettitt‐Wade et al. 2023). Although habitat shifts (*δ* ^13^C) were observed with increasing body length, these observed patterns suggest that body length is linked to foraging location but does not cause a change in trophic level feeding. Variation in environmental conditions across sampling years could impact habitat availability or use for feeding, but may still offer similar prey types, limiting the need for trophic specialization. Seasonal patterns were also observed in fish harvesting activity in Ulukhaktok, likely related to species life history and accessibility across land and waters for harvesting (Lea et al. 2023). Catchability associated with feeding rates and stable isotope samples can be paired with stomach content data to clarify individual feeding choices and specialization in prey items with size (Araújo et al. 2007) or seasonal variability (Cusa et al. 2019).

The observed intraspecific variation in Greenland cod indicates that individuals may exhibit variable responses to shifts in habitat and prey availability, such as those brought on by climate change effects. With community observations of decreased abundance of Greenland cod (Chan S., Personal Communication, July 2022), the western Arctic population may experience occurrences of overlap with sub‐Arctic species, with potential for competition and niche displacement of sub‐Arctic Gadids (Nielsen and Andersen 2001). The consequences of borealization can affect food web structure and prey availability (Florko et al. 2021; Fossheim et al. 2015), whereby generalists could adapt and shift their diet, while specialist individuals could be severely affected. However, Arctic generalists such as Greenland cod may be less impacted by competition as they would likely switch to alternative resources (Ma and Levin 2006). Arctic specialists that possess a narrower niche may avoid overlap, but be highly vulnerable in the event that their specialized niche experiences an environmental disturbance (Wilson et al. 2008). The cumulative impacts of increasing overlap with sub‐Arctic species and changes in prey availability could benefit or have minimal effect on generalist individuals, as the introduction of new prey types could increase prey diversity with little consequence on overall resource availability or abundance in their diet. For specialist individuals, the cumulative impacts could result in native prey being outcompeted, limiting their only food source. Our study revealed that Greenland cod morphotypes can be displayed along a generalist‐specialist gradient as a result of variable habitat‐feeding behaviors, and this may benefit this overall generalist species in the future, given their broad niche and ability to adapt to diverse resources.

Our findings provide an important contribution to our understanding of the association between morphological variation and generalist‐specialist resource use in a rarely studied coastal Arctic fish species. However, it is important to recognize the current limitations of geometric morphometrics, stable isotope analysis, and the lack of supporting literature for Greenland cod in the Arctic. For example, landmark identification from morphometrics is difficult in species with low color contrast in body features (such as Greenland cod), which led to the exclusion of many photographs from the current study as they did not pass our quality control for making precise landmarks. Diet‐tissue discrimination factors facilitate standardized comparison of stable isotope data by accounting for between‐tissue isotopic discrimination. We accounted for isotopic discrimination using a diet discrimination factor for leopard coral grouper due to similarities in morphology and behavior. When available, a discrimination factor for a more cold‐adapted species with a low metabolic rate would be preferable and may improve the precision of our IS estimates and facilitate comparison with other species and locations. Greenland cod is understudied, and this data deficiency is particularly evident in the Arctic, emphasizing the need for research that builds on recent advances in our understanding of this species' ecology and potential flexibility to food web shifts in the western Canadian Arctic.

## 5 Conclusion

As climate change continues to transform the marine ecosystem near Ulukhaktok and the Inuvialuit Settlement Region, the high variation in habitat‐diet metrics observed in Greenland cod and the gradient of generalist‐specialist behaviors may result in variable responses to environmental disturbances and may be a key factor contributing to their resilience. Where resources are inherently limited, the ongoing northward shift of sub‐Arctic species to the Arctic may favor endemic generalists that are capable of shifting their resource use to minimize niche overlap and competition. Future work should focus on understanding potential interactions between native Arctic species and closely related sub‐Arctic species. Our findings highlight the importance of maintaining trait variation to conserve diversity and promote resilience under a changing climate. We recommend that fisheries management and conservation strategies should be more inclusive toward population‐level trait diversity to promote adaptability and resilience to changing environmental conditions, especially for important subsistence species in the Canadian Arctic.

## Author Contributions

**Stephanie Chan:** conceptualization (equal), data curation (lead), formal analysis (lead), investigation (lead), methodology (equal), validation (lead), visualization (lead), writing – original draft (lead), writing – review and editing (equal). **Harri Pettitt‐Wade:** conceptualization (equal), data curation (supporting), formal analysis (supporting), funding acquisition (equal), investigation (supporting), methodology (equal), visualization (supporting), writing – review and editing (equal). **Jack P. W. Hollins:** conceptualization (equal), data curation (supporting), formal analysis (supporting), investigation (supporting), methodology (equal), validation (supporting), visualization (supporting), writing – review and editing (equal). **Tristan Pearce:** funding acquisition (equal), writing – review and editing (equal). **Lisa Loseto:** funding acquisition (equal), writing – review and editing (equal). **Teah G. Burke:** formal analysis (supporting), methodology (equal), visualization (supporting), writing – review and editing (equal). **Nigel E. Hussey:** conceptualization (equal), formal analysis (supporting), funding acquisition (equal), investigation (supporting), methodology (equal), supervision (lead), writing – review and editing (equal).

## Ethics Statement

Strict ethical guidelines were followed from the Canadian Council on Animal Care for the handling of live animals with approval from the University of Windsor Animal Care Committee; Reference #18–03, and Fisheries and Oceans Canada License to Collect Fish for Scientific Purposes; License # S‐19/20–3000‐YK. As guided by the Inuvialuit Game Council, the work was completed with full support and approval from the Olokhaktomiut Hunters and Trappers Committee (OHTC), and with collaborative feedback from the Ulukhaktok Char Working Group (UCWG).

## Conflicts of Interest

The authors declare no conflicts of interest.

## Data Availability

The data that support the findings of this study are openly available in GitHub at https://github.com/schan96/Greenland‐cod‐morphology‐SIA.git.

## Appendix A

**TABLE A1:** Contribution (%) of size‐adjusted linear measurements expressed as Principal Components (PC) toward explaining total variation of Greenland cod morphology.

PC axis	Eigenvalue	Percent variance (%)	Cumulative percent variance (%)
PC1	3.4	38.0	38.0
PC2	2.4	26.8	64.7
PC3	0.9	10.3	75.1
PC4	0.8	8.6	83.6
PC5	0.6	7.1	90.8
PC6	0.3	3.9	94.6
PC7	0.2	2.6	97.2
PC8	0.1	1.5	98.7
PC9	0.1	1.3	100.0
